# Mise à jour thérapeutique et pronostique de la rupture utérine dans une maternité à Bangui, CAR

**DOI:** 10.5588/pha.23.0004

**Published:** 2023-08-01

**Authors:** S. Huyghe, S. Telo, E. Danwesse, E. Ali, W. van den Boogaard, D. Lagrou, S. Caluwaerts, R. N. Ngbalé

**Affiliations:** 1 Médecins Sans Frontières Centre Opérationnel de Bruxelles, Mission en République Centrafricaine, Bangui, République Centrafricaine; 2 Ministère de la Santé, Direction de la Santé, Luxembourg, Luxembourg; 3 Médecins Sans Frontières Centre Opérationnel de Bruxelles, Luxembourg Operational Research Unit (LuxOR), Luxembourg, Luxembourg; 4 Médecins Sans Frontières Centre Opérationnel de Bruxelles, Département Médical, Bruxelles, Belgique; 5 Centre Hospitalier Universitaire Communautaire, Bangui, République Centrafricaine

**Keywords:** prévalence, facteurs associés, utérus cicatriciel, Afrique subsaharienne

## Abstract

**CONTEXTE:**

Le taux de mortalité maternelle reste élevé (882/100 000 naissances) en République Centrafricaine (RCA), du fait de la survenue de fréquentes complications obstétricales. Médecins Sans Frontières y soutient une maternité de référence à la capitale, Bangui.

**OBJECTIFS:**

Décrire la prévalence, les facteurs associés et la létalité, de l’une des plus sévères, la rupture utérine (RU), ainsi que l’influence d’un antécédent de chirurgie utérine.

**MÉTHODES:**

Ceci est une étude transversale sur des données collectées rétrospectivement entre janvier 2018 et décembre 2021 pour les femmes accouchées d’un nouveau-né plus de 1 000 g.

**RÉSULTATS:**

Sur 38 782 accouchements, 229 (0,6%) de RU étaient enregistrés. Les facteurs associés à la RU étaient : une parité ≥5 (ORb 7,5 ; IC 95% 4,6–12,2), une présentation fœtale non occipitale (ORb 2,8 ; IC 95% 2,1–3,7) et une macrosomie (OR 4 ; IC 95% 2,6–6,4). La létalité était de 4,4% et la mortinatalité de 64%. La RU était survenue sur utérus non cicatriciel chez 150 (66,1%) femmes. L’issue était plus défavorable en cas de survenue sur utérus non cicatriciel que cicatriciel avec plus de décès maternel (6% vs 0% ; *P = *0,023) et un Apgar du nouveau-né <2 (69,1% vs 45,8% ; *P < *0,001).

**CONCLUSION:**

La RU reste un problème majeur de santé maternelle et périnatale en RCA et des efforts sont nécessaires pour détecter précocement les facteurs de risque et d’augmenter la couverture des Soins Obstétricaux et Néonataux d’Urgence Complets.

## Abstract (en)

**BACKGROUND:**

Maternal mortality rates remain high (882/100,000 births) in the Central African Republic (CAR), primarily due to frequent obstetric complications. Médecins Sans Frontières supports a referral maternity ward in the capital, Bangui.

**OBJECTIVES:**

To describe the prevalence, associated factors and fatality of one of the most severe complications, uterine rupture, as well as the effect of a history of uterine surgery.

**METHODS:**

This is a cross-sectional study based on retrospectively collected data between January 2018 and December 2021 for women who delivered new-borns weighing over 1,000 g.

**RESULTS:**

Of 38,782 deliveries, 229 (0.6%) cases of uterine rupture were recorded. Factors associated with uterine rupture were parity ⩾5 (adjusted odds ratio [aOR] 7.5, 95% confidence interval [CI] 4.6–12.2), non-occipital foetal presentation (aOR 2.8, 95% CI 2.1–3.7) and macrosomia (OR 4, 95% CI 2.6–6.4). The fatality rate was 4.4%, and the stillbirth rate was 64%. Uterine rupture occurred in non-scarred uterus in 150 (66.1%) women. Adverse outcomes were more common in cases of uterine rupture on non-scarred uterus compared to scarred uterus, with higher maternal mortality (6% vs. 0%, *P* = 0.023) and lower Apgar scores (<2) for new-borns (69.1% vs. 45.8%, *P* < 0.001).

**CONCLUSION:**

Uterine rupture remains a major issue for maternal and perinatal health in the CAR, and efforts are needed to early detect risk factors and increase coverage of the comprehensive emergency obstetric and neonatal care.

La rupture utérine (RU) réalise une solution de continuité non chirurgicale aux dépens du myomètre ou de sa séreuse surtout au troisième trimestre de grossesse ou au moment du travail d’accouchement. Alors que la complication obstétricale sévère est devenue rare dans les pays à revenus moyens ou élevés, survenant chez moins de 0,1% des gestantes, elle reste relativement fréquente dans les pays à faibles revenus, avec une prévalence comprise entre 0,1% et 1%.^1^ Véritable urgence obstétricale, le taux de mortalité maternelle de la RU varie entre 1% et 13% et celui de la mortinatalité entre 74% et 92% selon les travaux.^1^

La RU peut survenir sur utérus cicatriciel ou non cicatriciel.^2^ L’absence de manifestations spécifiques rendant souvent le diagnostic difficile, cette urgence devrait être suspectée chez toute femme présentant un choc hémorragique et dont la cause n’est pas immédiatement évidente.^3^ Elle nécessite bien souvent des transfusions sanguines et une hystérectomie.^4,5^

Certains facteurs de risque de cette complication ont été rapportés dans la littérature. Il s’agit par exemple des antécédents de chirurgie utérine, d’une utilisation inadéquate des utérotoniques, d’un travail prolongé, d’accouchements dystociques, de la grande multiparité, des manœuvres obstétricales comme la version interne et plus rarement certaines malformations utérines congénitales.^2–4^ Le facteur de risque le plus important dans les pays à hauts revenus est un antécédent de césarienne ou une myomectomie, présent dans 80% des cas en moyenne, alors qu’ils ne sont retrouvés que dans 20% à 30% des cas dans certains pays d’Afrique subsaharienne,^1,6–10^ suggérant l’association possible d’autres facteurs de risque dans ces régions.

En République Centrafricaine (RCA), peu de données sont disponibles dans la littérature sur l’ampleur de la RU, les facteurs associés et son impact sur la santé materno-infantile. Les deux travaux antérieurs sur la question, retrouvés dans la littérature ont été menés dans l’hôpital de référence à Bangui, la capitale. Dans ces travaux qui n’analysaient pas les facteurs de risque, la prévalence retrouvée était de 0,6% dans l’un en 2002, avec 11,7% des cas survenus sur utérus cicatriciels.^11^ Dans le second, la prévalence rapportée en 2012 était de 0,3%, avec une mortalité maternelle de 21,4%.^12^

La maternité de Castors est située dans un centre de santé (CS) à Bangui. Elle est appuyée par Médecins Sans Frontières (MSF) depuis 2014 et met en œuvre des Soins Obstétricaux et Néonataux d’Urgence Complets (SONUC). Le présent travail vise à étudier les particularités de cette complication dans le contexte spécifique de cette maternité, en vue d’améliorer éventuellement les pratiques. En se basant sur les données colligées entre 2018 et 2021, les objectifs fixés étaient de : 1) décrire la prévalence de la RU et ses caractéristiques ; 2) identifier les facteurs qui lui étaient associés ; 3) déterminer la mortalité maternelle et la mortinatalité ; et 4) comparer les particularités du tableau clinique et l’issue selon l’existence ou non d’un antécédent de chirurgie utérine.

## MÉTHODES

### Type d’étude

Il s’agissait d’une étude transversale, descriptive et analytique avec collecte rétrospective des données.

### Cadre

#### Cadre général

La RCA a l’un des taux de mortalité maternelle les plus élevés au monde (882 décès/100 000 naissances), et une parité élevée de 4,6 naissances par femme en 2019.^13,14^ Le taux de mortalité néonatale est également très élevé, 39,7/1 000 naissances vivantes.^15^ Le pays est confronté à une pénurie de personnel qualifié dans les services de santé sexuelle et reproductive. Le ratio de sage-femmes/infirmiers pour 1 000 personne est de 0,206 (10/1 000 aux pays à haut revenu).^16^ De plus, leur accessibilité est limitée du fait du conflit militaro-politique qui sévit dans le pays depuis plusieurs années.^17^

#### Cadre spécifique

Le CS de Castors qui abrite la maternité est situé dans le troisième arrondissement de la ville de Bangui. Vue la gratuité des soins, il exerce une grande attractivité sur les patients vivant dans les régions environnantes. Sur le plan logistique, le centre abrite entre autres un bloc opératoire et une banque de sang, et est capable d’administrer des soins néonataux intensifs. Une moyenne de 900 accouchements était réalisée à la maternité par mois.

### Population de l’étude

Toutes les femmes admises à la maternité des Castors entre 2018 et 2021, ayant accouché d’un nouveau-né plus de 1 000 g ont été inclus.

### Collecte de données

Les informations sur les variables étudiées ont été extraites de la base de données de surveillance routinière du projet MSF. De plus, les dossiers médicaux personnels physiques ont été exploités par trois enquêteurs, afin de valider les cas de RU et de recueillir des informations additionnelles sur une fiche préparée pour l’étude. Ces informations ont été ensuite doublement saisies par deux encodeurs de façon pseudonymisé dans un fichier électronique. Les deux bases de données ont été fusionnées pour créer la base de données MS Excel (Microsoft, Redmond, WA, États Unis) utilisée pour les analyses.

### Analyse statistique

Les données étaient transférées anonymisé dans le logiciel de STATA v16 (Stata Corp, College Station, TX, États Unis) pour faire les analyses. Les variables catégorielles ont été décrites en utilisant les proportions et leur intervalle de confiance à 95% (IC 95%) ; les variables quantitatives avec la médiane, son intervalle interquartile (IQR) pour les variables continues. Les facteurs associés à la RU ont été recherchés par régression logistique simple, avec détermination de l’*odds ratio* (OR), son IC 95% et la valeur *P*. Des comparaisons ont été faites entre les femmes ayant eu une RU, selon la présence ou non d’un utérus cicatriciel, en utilisant le test du χ^2^ (ou le test exact de Fisher si applicable). Le seuil de significativité était fixé à 5%.

### Considérations éthiques

L’approbation du Comité Ethique et Scientifique de la RCA, Bangui, RCA, a été préalablement obtenue (N°_38_/UB/FACSS/IPB/CES/022). Cette recherche remplissait les critères d’exemption fixés par le Comité d’éthique de MSF pour les analyses a posteriori des données cliniques collectées de manière routinière et ne nécessitait donc pas d’examen MSF Ethics Review Board. Elle a été menée avec l’autorisation du directeur médical du Centre Opérationnel de Bruxelles, Belgique.

## RÉSULTATS

Dans la maternité de Castors, de 2018 à 2021, 229 cas de RU ont été enregistrés sur 38 782 accouchements réalisés, soit une prévalence de 0,6%. Elle est passée de 43,1 en 2018 à 74,2 par 10 000 accouchements en 2021 (Figure). L’âge médian était 29 ans (IQR 24–32). Parmi elles, 137 (59,8%) étaient référées d’une autre structure, et plus précisément, 22 (9,6%) d’un autre centre SONUC ; 70 (30,8%) n’avaient jamais eu de consultation prénatale ; 51 (22,5%) avaient une parité supérieure ou égale à 5 (Tableau 1). Après régression logistique simple, les femmes référées d’un centre SONUC (ORb 12,9 ; IC95% 7,9–20,4), d’un centre soins obstétricaux et néonataux d’urgence de base (ORb 10,2 ; IC 95% 7,5–13,9) ou d’une autre structure sanitaire (ORb 9,8 ; IC 95% 6,6–14,3) étaient plus à risque d’avoir eu une RU que celles venues spontanément de leur domicile. Les autres facteurs liés à la femme ou au nouveau-né qui étaient associés à la RU étaient une parité comprise entre 1 et 4 (ORb 3,5 ; IC 95% 2,3–5,3), ou au-delà pour les grandes multipares (ORb 7,5 ; IC 95% 4,6–12,2), une présentation du fœtus autre qu’occipitale (ORb 2,8 ; IC 95% 2,1–3,7), et une macrosomie, c’est-à-dire un poids de naissance au-delà de 4 000 g (ORb 4,1 ; IC 95% 2,6–6,4) (Tableau 2). Parmi les caractéristiques cliniques et thérapeutiques, la RU était survenue avant l’admission chez 138 (73,8%) femmes ; 206 (90,3%) n’avaient pas reçu d’utérotoniques ; et 130 (59,9%) étaient dans la période active du travail au moment du diagnostic. Le délai médian entre l’admission et le diagnostic était de 39 min (IQR 14–246) ; et celui écoulé entre le diagnostic et l’intervention de 32 min (IQR 18–47). Dans 152 (67,9%) cas, une hystérorraphie sans recours à une hystérectomie était réalisée (Tableau 3).

**FIGURE:**
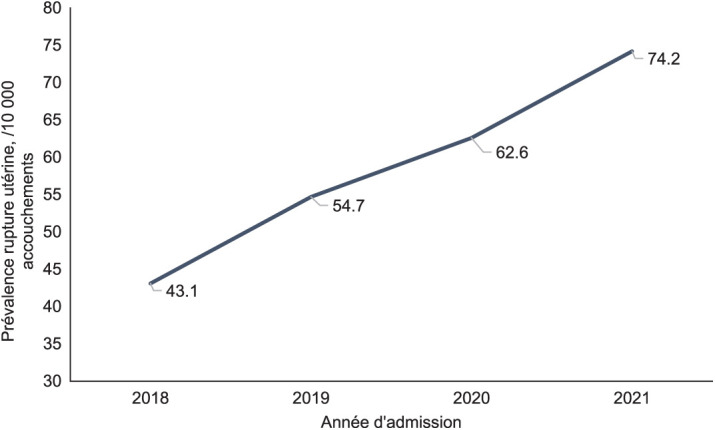
Evolution de la prévalence de rupture utérine (par 10 000 accouchements) à la maternité de Castors à Bangui, République Centrafricaine, entre 2018 et 2021.

**TABLEAU 1:** Caractéristiques des femmes avec rupture utérine prises en charge à la maternité de Castors à Bangui, République Centrafricaine, entre 2018 et 2021

	*n*	(%)
Type de référence (*n* = 229*)		
Spontanée	92	(40,2)
SONUB	77	(33,6)
SONUC	22	(9,6)
Autre^†^	38	(16,6)
Consultations prénatales (*n* = 227*)		
Non	70	(30,8)
Oui	157	(69,2)
Parité, *n* (*n* = 227)		
0	24	(10,6)
1–4	152	(67,0)
⩾5 (grande multiparité)	51	(22,5)
Age gestationnel en semaine d’aménorrhée (*n* = 197*)		
>36	160	(81,2)
<36	37	(18,8)
Mode d’accouchement (*n* = 212^*^)		
Accouchement normal	47	(22,2)
Ventouse	8	(3,8)
Césarienne	157	(74,1)
Type de présentation du fœtus (*n* = 211*)		
Occipital	131	(62,1)
Non occipital^‡^	80	(37,9)
Poids de naissance du nouveau-né en grammes (*n* = 210*)		
1 000–2 000	8	(3,8)
2 000–3 000	56	(26,7)
3 000–4 000	124	(59)
⩾4 000	22	(10,5)

*Total avec information disponible.

^†^Structures sanitaires non identifiées comme SONUC ou SONUB par Médecin Sans Frontières à (ou hors de) Bangui.

^‡^Autre présentation que la présentation occipitale, dans laquelle le sommet n’est la partie la plus engagée (présentation du front, du face, siège, position transversale, de l’épaule…) ;

SONUB = soins obstétricaux et néonataux d’urgence de base ; SONUC = Soins Obstétricaux Et Néonataux D’urgence Compréhensive.

**TABLEAU 2:** Facteurs associés après régression logistique simple à la rupture utérine chez les femmes prises en charge à la maternité de Castors à Bangui, République Centrafricaine, entre 2018 et 2021

	Total	Rupture utérine		ORb	(IC 95%)	*P*-value
		*n*	/10 000			
Provenance						
Domicile	33 831	92	27,2	Référence		
SONUB^¤^	2 843	77	270,8	10,21	7,51–13,85	<0,001
SONUC^¤¤^	649	22	339,0	12,86	7,87–20,36	<0,001
Autres*	1 459	38	260,5	9,81	6,63–14,29	<0,001
Consultations prénatales						
Oui	28 357	157	55,4	Référence		
Non	10 413	70	67,2	1,22	0,92–1,61	0,176
Parité						
0	12 406	24	19,3	Référence		
1–4	22 797	152	66,7	3,46	2,25–5,33	<0,001
⩾5	403	51	1 265,51	7,47	4,59–12,16	<0,001
Type de présentation du foetus						
Occipital	31 699	131	41,3	Référence		
Non occipital^†^	6 920	80	115,6	2,81	2,13–3,73	<0,001
Poids de naissance du bébé, g						
<4 000	37 492	188	50,1	Référence		
⩾4 000	1 088	22	202,2	4,09	2,62–6,39	<0,001

*Autres structures sanitaires non identifiées comme SONUC ou SONUB par Médecins Sans Frontières à (ou en dehors de) Bangui.

^†^Autre présentation que la présentation occipitale, dans laquelle le sommet n’est la partie la plus engagée (présentation du front, du face, siège, position transversale, de l’épaule…).

^¤^SONUB = soins obstétricaux et néonataux d’urgence de base ; ­SONUC = soins obstétricaux et néonataux d’urgence compréhensive; ORb = *odds ratio* brut ; IC 95% = intervalle de confiance à 95%.

**TABLEAU 3:** Caractéristiques cliniques et thérapeutiques des femmes avec rupture utérine prises en charge à la maternité de Castors à Bangui, République Centrafricaine, entre 2018 et 2021

	*n*	%
Rupture utérine avant l’admission (*n* = 227)		
Non	49	26,2
Oui	138	73,8
Phase du travail au moment du diagnostic (*n* = 227*)		
Phase de latence	60	27,7
Phase active	130	59,9
Phase postpartum	27	12,4
État hémodynamique (*n* = 228*)		
Stable	175	76,7
Choc	53	23,5
Partogramme (*n* = 219*)		
Non	25	11,4
Oui	46	21,0
Non applicable	148	67,6
Utilisation des utérotoniques^†^ (*n* = 228*)		
Non	206	90,3
Oui	22	9,7
Type de rupture utérine (*n* = 227*)		
Déhiscence	40	17,6
Complète	187	82,4
Localisation de la rupture utérine (*n* = 226*)		
Transverse - segment inferieur	80	35,4
Corporel	28	12,4
Fundique	13	5,7
Combiné^‡^	105	46,5
Technique chirurgicale (*n* = 224*)		
Hystérorraphie	152	67,9
Hystérectomie	72	32,1
Transfusion (*n* = 227*)		
Non	104	45,8
Oui	123	54,2

*Total avec information disponible.

^†^Misoprostol, ocytocine.

^‡^Rupture suivant plusieurs axes.

A l’issue de la prise en charge, le taux de létalité était de 4,4% et 0,1%, respectivement, chez les femmes avec et sans RU (*P = *0,234), restant stable chez les femmes avec RU durant les 4 années (entre 4,3% et 5,3%). Concernant le bébé, la mortinatalité était de 64% en cas de RU et de 3,6% en l’absence de cette complication (*P *< 0,001).

En s’intéressant spécifiquement aux antécédents de chirurgie utérine, l’information était disponible chez 227 femmes sur les 229. La RU était survenue sur un utérus non cicatriciel chez 150 (66,1%) et sur un utérus cicatriciel chez 77 (33,9%, des anciennes césariennes et une seule myomectomie). Chez les femmes avec RU sur utérus cicatriciel, le temps écoulé depuis la césarienne précédente était inférieur à 24 mois chez 19% ; et elles avaient plus souvent une rupture transverse sur le segment inférieur comparées à celles ayant un utérus non cicatriciel (62,3% vs 21,6% ; *P < *0,001). A l’opposé, les femmes avec RU sur utérus non cicatriciel, avaient significativement plus souvent une localisation corporéale (16,2% vs 5,2% ; *P = *0,021), fundique (8,1% vs 1,3% ; *P = *0,038) ou combinée (54,1% vs 31,2% ; *P = *0,014). Elles étaient plus souvent en état de choc hémodynamique (28,2% vs 11,7% ; *P = *0,010), avec un besoin transfusionnel accru (63,1% vs 36,4% ; *P < *0,001) ; le recours à une hystérectomie était plus fréquent (40,8% vs 14,5% ; *P < *0,001) ; de même qu’un score Apgar du bébé inférieur à 2, (69,1% vs 45,8% ; *P < *0,001) (Tableau 4).

**TABLEAU 4:** Comparaison des caractéristiques cliniques, thérapeutiques et du devenir de la femme avec rupture utérine et du bébé selon la présence ou non d’un utérus cicatriciel à la maternité de Castors à Bangui, République Centrafricaine, entre 2018 et 2021

	Utérus non cicatriciel *n *(%)	Utérus cicatriciel *n* (%)	*P*-value*
Total	(*n* = 150 ; 66,1%^†^)	(*n* = 77 ; 33,9%^†^)	
Mère décédée, *n*	149*	77^*^	
Non	140 (94)	77 (100)	0,657
Oui	9 (6)	0	0,023
Score Apgar du bébé, *n*	139*	72*	
⩾7	31 (22,3)	34 (47,2)	0,003
2–6	12 (8,6)	5 (6,9)	0,709
<2	96 (69,1)	33 (45,8)	0,038
Localisation du rupture utérine, *n*	148*	77*	
Corporel	24 (16,2)	4 (5,2)	0,021
Transverse-segment inférieur	32 (21,6)	48 (62,3)	<0,001
Fundique	12 (8,1)	1 (1,3)	<0,038
Combiné^‡^	80 (54,1)	24 (31,2)	0,014
Etat hémodynamique, *n*	149*	77*	
Etat stable	107 (71,8)	68 (88,3)	0,185
Choc	42 (28,2)	9 (11,7)	0,010
Transfusion, *n*	149*	77*	
Non	55 (36,9)	49 (63,6)	0,006
Oui	94 (63,1)	28 (36,4)	<0,001
Type de chirurgie, *n*	147*	77*	
Hystérorraphie	87 (59,2)	65 (85,5)	0,032
Hystérectomie	60 (40,8)	11 (14,5)	<0,001

*Total avec information disponible sur la variable.

^†^Dénominateur = 227 (information non disponible chez 2 femmes sur les 229).

^‡^Rupture suivant plusieurs axes.

## DISCUSSION

Il s’agit de l’une des rares études menées sur la RU dans un centre SONUC en RCA. Entre 2018 et 2021, la prévalence de la RU était de 6 pour 1 000 accouchements ; et le nombre de cas a presque doublé. Six femmes sur 10 provenaient d’une autre structure sanitaire, et près d’une personne sur 10 d’un autre centre SONUC. Au nombre des facteurs associés à une RU, il y avait par ordre d’importance, une grande multiparité, une macrosomie, une présentation non occipitale. Après prise en charge, la létalité était de 44 pour 1 000 femmes avec RU et était assez stable dans le temps ; par contre, la mortinatalité était très élevée. En effet, plus de six bébés sur 10 étaient décédés en cas de RU, alors moins d’un bébé sur 10 l’était en l’absence de cette complication. Lorsque la rupture survenait sur un utérus non cicatriciel, les complications maternelles étaient plus fréquentes et le pronostic du fœtus plus sombre qu’en cas d’utérus cicatriciel.

La prévalence de la RU retrouvée dans ce travail mené dans un CS est comparable à celle rapportée d’autres pays d’Afrique subsaharienne en milieu hospitalier.^8,10,18^ Des prévalences plus élevées ont été rapportées dans certaines études en Ethiopie, entre 2% et 4%.^7,9^ A Bangui, des travaux précédents réalisés à l’hôpital communautaire ont retrouvé une tendance à la diminution de la prévalence de la RU entre 2002 et 2012,^11,12^ contrastant avec l’augmentation constatée dans la présente étude. Les raisons ne sont pas très connues mais pourraient être en rapport avec une amélioration de l’enregistrement des cas ou une augmentation des cas référés. La pandémie de COVID-19 en 2020 ne semble pas avoir constitué un obstacle à l’accès au CS de Castors puisque le nombre d’admissions a continué à augmenter (10 544 en 2020 contre 10 418 en 2019 et 8 345 en 2018) De plus, la RCA a connu un impact généralement limité de COVID-19 avec 113 de morts sur 15 367 cas depuis le début de la pandémie.^19^

Contrairement à l’augmentation de la prévalence, la faible létalité constante est rassurante, et contraste avec les résultats d’autres travaux dans la littérature, où elle pouvait concerner une femme sur sept, voire sur cinq.^12,18^ Une explication possible est la prise en charge dans un centre SONUC, dont les caractéristiques comme un ratio personnel/patient élevé, une disponibilité d’une banque de sang avec un stock suffisant, et une formation continue du personnel soignant, améliorent la performance.

En s’interrogant sur la provenance de ces patientes, six femmes sur 10 avec RU ont été référées d’une autre structure sanitaire. Ces résultats corroboraient ceux d’autres auteurs au Sénégal, où les patientes référées étaient 2,56 fois plus susceptibles d’avoir une RU que celles venues de leur domicile.^20^ De façon surprenante, pour des raisons non clairement élucidées, une femme sur 10 provenait d’un autre centre SONUC de la ville, qui, théoriquement, offrent les mêmes services de la prise en charge que celui appuyé par MSF. Des échanges informatives et pratiques sur les modalités de la prise en charge dans ces structures pourraient aider à améliorer la situation.

Au nombre des facteurs associés, le risque de RU était 7,5 fois plus élevé chez les femmes avec une grande multiparité (≥5), comparées aux nullipares, certainement en lien avec un utérus plus fibreux et moins tonique. Les mêmes résultats ont été retrouvés dans l’étude QUARITE menée au Mali et au Sénégal, avec un OR de 7,5.^8^ Il en est de même d’autres travaux aux pays à faible revenu dans la littérature qui ont aussi démontré une association entre la RU et la parité.^7,9,21^ Les autres facteurs de risque retrouvés comme la présentation non occipitale et la macrosomie ont aussi été rapportés par d’autres auteurs au Sénégal et à Madagascar.^20,22^ Les contractions plus prolongées et la forte sollicitation du muscle utérin dont ils sont responsables facilitent sans doute la RU.

Contrairement à des données publiées ailleurs,^9^ aucune différence n’était notée entre la non-participation aux consultations prénatales et la survenue d’une RU. Pour autant, elles doivent être encouragées, étant donné qu’elles constituent des opportunités de dépistage précoce des facteurs de risque de RU.^23^

Par ailleurs, l’étude s’est intéressée spécifiquement à l’impact d’un utérus cicatriciel sur le pronostic materno-fœtal. Le défaut d’encodage systématique de cet antécédent chez toutes les femmes admises au CS de Castors n’a pas permis d’investiguer une éventuelle association avec une RU dans le présent travail. Elle a pu être possible dans le groupe spécifique des femmes avec une RU. La fréquence nettement plus élevée (7/10) d’un utérus non cicatriciel parmi les femmes avec RU a aussi été rapportées par d’autres auteurs d’Afrique subsaharienne.^7–10^ De même, Gibbins et al. ont aussi constaté une fréquence plus élevée de complications maternelles et un pronostic fœtal plus défavorable chez ces patientes.^24^ Les localisations corporéales, fundiques ou combinées multidirectionnelles plutôt que transversales du segment inférieur, plus fréquentes chez ces patientes avec utérus non cicatriciel, pourraient expliquer ce pronostic plus péjoratif, vu le risque plus élevé de lésions vasculaires et d’hémorragie intra-abdominale.^25^ Ces cas nécessitent un plateau technique plus performant, souvent absent dans les pays à faibles revenus.

La principale limite de ce travail est relative aux données manquantes sur certaines variables étudiées, vu sa nature rétrospective. Il s’agit en particulier du délai entre le début du travail et l’heure d’arrivée à Castors, avec un probable impact sur la survenue et l’issue d’une RU. Dans la mesure du possible, elles ont été complétées à partir des dossiers physiques. Ses forces résident entre autres dans la grande taille de la population et la prise en compte de toutes les femmes, permettant ainsi de minimiser les biais liés aux fluctuations d’échantillonnage. De même, cette étude a permis d’apprécier l’évolution de cette urgence obstétricale sur quatre années.

Enfin, certaines implications opérationnelles pourraient en découler. En effet, il reste important de former les professionnels impliqués dans les soins obstétricaux sur les facteurs associés à la RU, qui ont été mis en évidence, pour une référence précoce de parturientes à risque vers un centre SONUC, mieux outillé pour prévenir le développement d’une RU. Ensuite, il faut plus de recherche sur les retards éventuels avant l’arrivée au SONUC et ses facteurs déterminants. Finalement, fort de la faible létalité constatée dans le SONUC de Castors, des efforts supplémentaires doivent être consentis pour améliorer la couverture des soins SONUC, et progressivement atteindre la cible de l’OMS d’un SONUC pour 500 000 personnes.^26^ Ces implications sont conformes à celles des revues de décès maternels en Ouganda, en mettant l’accent sur des soins obstétriques précoces et adéquats pour les patientes à haut risque d’une RU.^27^

En conclusion, cette étude a montré que la RU, spécifiquement, celle survenant sur utérus non cicatriciel, continue de menacer la santé maternelle et périnatale chez les femmes en RCA. Certains facteurs associés à la RU ont été identifiés, dont la prise en compte devrait prévenir la survenue et l’issue de cette urgence.

### Remerciements

Les auteurs remercient B Capelli, référent médical du projet MSF Castors, Bangui, RCA, au moment de l’étude et toute l’équipe médicale de cette maternité ; J Gil et T Songo-Kette Gbekere pour sa contribution à l’écriture du protocole, A Acma pour son support dans la collecte des données ; et A Rakesh et D Obach dans l’analyse des données.

Cette recherche a été menée dans le cadre d’une initiative de formation structurée en recherche opérationnelle (SORT-IT), un partenariat mondial dirigé par le Programme Spécial de Recherche et de Formation sur les Maladies Tropicales de l’OMS (OMS/TDR). Le modèle est basé sur un cours élaboré conjointement par l’Union Internationale contre la Tuberculose et les Maladies Respiratoires (L’Union) et Médecins Sans Frontières (MSF/Doctors Without Borders). Le programme spécifique, SORT-IT, qui a donné lieu à cette publication a été organisé par MSF spécifiquement pour la recherche en langue française. Le programme a été financé par La Fondation Veuve Emile Metz-Tesch, Luxembourg. Le financeur n’a joué aucun rôle dans la conception de l’étude, la collecte et l’analyse des données, la décision de publier ou la préparation du manuscrit.
